# Pyorrhea Alveolaris*An informal talk before the Research Society of New Jersey, reprinted from “The New Jersey Dental Journal.”

**Published:** 1918-01

**Authors:** Arthur H. Merritt

**Affiliations:** New York


### PYORRHEA ALVEOLARIS *

BY ARTHUR H. MERRITT, D.D.S., NEW YORK

There is no problem in the domain of dental practice more worthy of study than that of pyorrhea, inasmuch as it is a disease responsible for the loss of more teeth among that portion of the public that patronizes the dentist than any other found in the mouth; and it is responsible also for a vast number of secondary infections, involving the health, efficiency and well being of the individual. It is not extravagant to speak of it as the king of mouth infections. It may be defined as a progressive resorption of the alveolar process and preicementum with a coincident recession and inflammation of the gingiva and the formation

* An informal talk before the Research Society of New Jersey, reprinted from "The New Jersey Dental Journal."

of pockets along the roots of affected teeth. It is a disease of the investing tissues of the teeth, not of the teeth themselves. It has for its etiology a variety of conditions which may be divided into predisposing and exciting, such as lowered resistance, frail bony support of teeth, localized malnutrition, malocclusion, occlusal trauma, salivary deposits, filth, faulty contact points, parasitic invasion, ill-fitting crowns, bridges, fillings, etc. In a word, any condition causing gingival irritation. It is not by any one of these, but by a combination of them that pyorrhea is caused. Of these the most common predisposing cause, and the one most frequently overlooked, is occlusal trauma, a malrelation of the occlusal planes of the teeth when in use whereby an excessive strain is imposed upon one or more of them, of such a nature as to actually induce pathological changes in their investment.

Teeth may be in normal occlusion according to the Angle classification and at the same time be the subject of occlusal trauma. This explains why it is that one so frequently sees pyorrhea associated with so called normal occlusion. The absence of wear or cuspal abrasion interferes with the gliding motion of tooth against tooth when brought into occlusion whereby the stress of mastication is to a certain extent dissipated.

The result is, this strain is communicated to the tissue surrounding the roots and manifested in a thickening of the pericementum, rarefaction of the bone, and loosening of the affected teeth. These changes may all take place without solution of continuity in the floor of the subgingival space, indicating occlusal mal-adjustment, which if not corrected will probably result in loss of the teeth through the development of pyorrhea.

Among existing causes, the most common is gingival irritation. This may be caused by salivary deposits, filth, faulty contact points, ill-fitting crowns, etc. Whatever its cause, it should always be seriously considered for it is at this point that pyorrhea has its beginnings. Every inflamed gingiva should be carefully examined and its cause removed, in an effort to prevent pyorrhea, for it can be prevented more easily than any disease occurring in the mouth.

Regarding the bacteriology of pyorrhea, it can be said that at present there is no evidence that it is caused by a specific bacterium; on the contrary there is plenty of evidence that it is not. Studies of the bacterial flora of pyorrhea have shown that it does not differ qualitatively from that of the normal mouth. As might be expected there is a great quantitative difference inasmuch as pyorrheal conditions are conductive to bacterial growth. Investigations carried on at the Research Laboratories of the New York Health Department through my initiative have proven this beyond question. It showed, moreover, the extraordinary complexity of the bacterial flora of pyorrheal lesions, Spirochetes and fusilform bacilli preponderated. No less than fifteen different strains of the latter were isolated and carefully studied. Nothing distinctive of pyorrhea was found. Cocci of every known strain were found as were many other morphological types. Comparisons with cultures taken from normal mouth were made only to find that the same organisms were to be found, only in greatly reduced number, showing the difference to be purely a quantitative one. The use, therefore, of vaccines in the treatment of pyorrhea is illogical in the extreme. The same may be said of emetin. While it is an efficient amebacide, and will destroy amebas which are always present in pyorrheal lesions (as they are in every filthy mouth), it has no curative effect.

Pyorrhea will never be cured by the thousand and one nostrums so plausibly advocated. It is, however, a curable disease, all statements to the contrary notwithstanding. This can not be made too emphatic. The day is past when the dentist can tell his patient that pyorrhea is an incurable disease, and get away with it. Too many men are successfully treating it to make that statement a safe one. The first requisite to success is the correction of any occlusal trauma which may exist. This is done by judicious grinding of the inclined planes of all teeth shown to be involved. Test all teeth with thin carbon paper and so grind the contact points as to permit of greater freedom of motion. Small carborundum stones, such as those made by Miller, are best adapted for this work. The grinding should be done in such a way as to relieve without disfiguring the teeth. Indiscriminate grinding is unwise. The second essential and the one most difficult to achieve is good root surgery. This means the *complete removal* from the surface of the exposed cementum, of all calcareous deposits and the necrotic pericementum, care being exercised not to cut through into the dentin, at the same time leaving the root surface smooth and polished. This is not easily done, and with the ill-assorted heterogenous instruments so often employed, it is wholly impossible. The dental profession has not yet grasped the importance of correct instruments, it is still laboring under the delusion that any old instrument will do with which the grosser deposits can be removed, regardless of how many grooves and scratches it may inflict upon the surface of the cementum. Of first importance therefore to good root surgery is proper instruments. Of these there are two general types—planes and curettes. The latter is the type in more general use in the east, and comprises those designed by Younger, Good and Hutchinson. These sets are fewer in number and less complicated than are the planes. They are designed to reach the different surfaces of exposed cementum, and to remove from it all calcareous deposits as well as dead pericementum, at the same time to leave the root smooth. A grooved and roughened surface is prejudicial to good results. To leave a finished surface for physiological contact with the adjacent soft tissues is essential before union between them can be expected. When properly done such union takes place on vital teeth when other conditions are favorable. This means re-attachment and obliteration of pockets with re-establishment of health in the involved tissues. This never occurs in cases of non vital teeth which have become septic. Nor does it, always occur about vital teeth, due in most instances, no doubt, to faulty technique, though low recuperative power and inability to control certain conditions doubtless explains some failures. Of great importance in the care of these cases is intelligent post-operative care by the patient. This means the maintenance of a high order of mouth hygiene, supplemented by such dental care as may be required to keep the mouth clean and the gingiva free from irritation. This is not easily achieved, as many patients do not intelligently cooperate in doing their part, which in reality is the more important, inasmuch as they have the daily care of their mouths. Therefore, it is necessary to carefully instruct them by demonstrations of correct procedures in one's own mouth. Any method to be satisfactory must be simple, efficient and stimulating to gums. It must accomplish this without injury to the mouth or teeth; which is impossible if horizontal brushing is indulged in. Personally I have found the up and down method most satisfactory as it meets every requirement—is simple, efficient and exceedingly stimulating to the gums and subjacent tissues. This is not to say that there are not other methods or to quarrel with any of those that may advocate them. A small brush of good quality is the first requisite. Dentrifices of known composition should be prescribed for their saponifying and abrasive effect. These have no therapeutic action worthy of consideration. Mouth washes are of little value except possibly for their soothing effect while the patient is under treatment. They have little value in the post operative treatment. Waxed silk, and in some instances porte polishers should be placed in the hands of patients with instructions as to their proper use.

To summarize briefly it may be said that pyorrhea is a curable disease though it is easier and far more satisfactory to prevent it; that it has a variety of causes, chief among them being gingival irritation and spongy and poorly nourished gums; that treatment consists in good root surgery and such attention to the occlusions as may be required, including intelligent home care by the patient.

